# Red Cell Distribution Width as a Prognostic Factor and Its Comparison with Lactate in Patients with Sepsis

**DOI:** 10.3390/diagnostics11081474

**Published:** 2021-08-14

**Authors:** Tsung-Han Wang, Yin-Chou Hsu

**Affiliations:** 1 Department of Emergency Medicine, E-Da Hospital, I-Shou University, Kaohsiung 82445, Taiwan; ch277652@yahoo.com.tw; 2 School of Medicine for International Student, I-Shou University, Kaohsiung 82445, Taiwan; 3 School of Chinese Medicine for Post Baccalaureate, I-Shou University, Kaohsiung 82445, Taiwan

**Keywords:** sepsis, erythrocyte indices, lactate, prognosis

## Abstract

Sepsis remains the leading cause of death in critically ill patients. Thus, regular measurement of lactate levels has been proposed in sepsis guidelines. Elevated red cell distribution width (RDW) is associated with mortality risk in patients with sepsis. This study aimed to investigate the association between RDW and the risk of other adverse outcomes in patients with sepsis and to compare the mortality discriminative ability between lactate and RDW levels. This is a single-centered, retrospective, case-control study that included 504 adult patients with sepsis in the emergency department between 1 January 2020 and 31 December 2020. Eligible patients were divided into normal (RDW ≤ 14.5%) and high (RDW > 14.5%) groups. The baseline characteristics and adverse outcomes were recorded and compared. Compared with the normal RDW group, the patients in the high RDW group had a significantly higher rate of ICU admission (48.8% vs. 32.4%, *p* = 0.03), septic shock (39.2% vs. 23.5%, *p* < 0.01), and 30-day in-hospital mortality (32.0% vs. 20.7%, *p* < 0.01). Furthermore, the RDW (area under curve (AUC) = 0.71) had superior mortality discriminative ability compared to lactate (AUC = 0.63) levels (*p* = 0.02). Clinicians could rely on this simple and rapid parameter for risk stratification to initiate prompt treatment for patients with sepsis.

## 1. Introduction

Sepsis is a status of organ dysfunction caused by a dysregulated host response to infection and is a leading cause of mortality and morbidity worldwide [1]. An updated practice guideline proposed by the Surviving Sepsis Campaign (SSC) recommended that serum lactate measurement is vital for patients with sepsis and septic shock, as increased levels may represent tissue hypoxia or other life-threatening scenarios [2]. However, conventional lactate measurement in a central laboratory is a time-consuming process that requires proper handling and is not readily available in all institutions [3]. A point-of-care test is an emerging method to measure lactate more efficient, although significant variance exists [4].

Red cell distribution width (RDW) represents the variability in size and form of all circulating red blood cells (RBCs) [5]. It is calculated based on the standard deviation of the RBC volume divided by the mean corpuscular volume (MCV) [6]. RDW is a usual laboratory parameter used as a tool in the diagnosis of different causes of anemia [5]. Elevated RDW was proposed to be associated with inflammatory processes and oxidative stress increase, resulting in the suppression of RBC maturation and release of large premature RBCs [7,8]. Recent studies have investigated the clinical utility of RDW in prognostic stratification in patients with sepsis and septic shock, and demonstrated that high RDW is associated with an increased risk of mortality in patients with sepsis [9,10,11].

Notably, few studies have compared the prognostic ability of RDW and lactate levels in patients with sepsis [12]. In addition, most RDW studies on patients with sepsis focused on mortality risk, and rarely discussed other adverse outcomes, such as risk of septic shock, respiratory failure, or intensive care unit (ICU) admission. The goal of this study is to explore the association between RDW and the risk of adverse outcomes in patients with sepsis and to compare the mortality discriminative ability between lactate and RDW levels.

## 2. Materials and Methods

### 2.1. Study Design

An observational case-controlled study was conducted retrospectively at a tertiary referral medical center in southern Taiwan that receives approximately 59,000 emergency department (ED) visits per year. All adult patients (aged ≥ 18 years) who visited the ED between 1 January 2020 and 31 December 2020 that underwent blood culture tests and received intravenous antibiotics were enrolled. The diagnosis of sepsis was further confirmed by ED diagnostic codes (International Classification of Disease, 10th revision, ICD-10) and medical records. Sepsis treatment followed the SSC guidelines [2]. Patients with known hematological disease (e.g., leukemia, myelodysplastic syndrome), human immunodeficiency virus (HIV) infection, immunosuppressant use, a cardiac arrest event before ED arrival, no lactate measurement, or who had recent (less than one week) blood transfusion, were excluded. The study protocol followed the STROBE guidelines [13], and was approved by the local institutional review board (EMRP-109-012). Requirement for informed consent was waived because of the retrospective observational nature of the study.

### 2.2. Data Collection

Baseline characteristics, sepsis source, comorbidities, and laboratory results of eligible patients were collected from anonymized electronic medical records. Laboratory results were obtained for each patient at the time of their initial ED visit. RDW was measured as a part of the complete blood count panel, and the reference range was 11.5–14.5% in our institution. Patients were categorized into normal (RDW ≤ 14.5%) and high (RDW > 14.5%) groups for further analysis. The presence of frailty syndrome was evaluated by daily progress notes of each patient. Comorbidities were based on disease codes. The source of sepsis was classified as follows: respiratory tract infection (radiological increased infiltration combined with clinically compatible symptoms), urinary tract infection (urinalysis revealed pyuria and bacteriuria), intra-abdominal infection, soft tissue infection, and others, in accordance with the discharge diagnosis of each patient.

### 2.3. Definitions

The systemic inflammatory response syndrome (SIRS) criteria were defined according to previous consensus [14]. The quick sepsis-related organ failure assessment (qSOFA) score was determined using ED triage parameters: Glasgow coma score < 15, respiratory rate ≥ 22 breaths/min, and systolic blood pressure ≤ 100 mmHg [1]. The sepsis-related organ failure assessment (SOFA) score was calculated based on the worst variables of organ dysfunction recorded within 6 h of sepsis recognition [15]. The diagnosis of frailty syndrome was established in the presence of 3 of the 5 components: fatigue, resistance, aerobics, illness, or loss of weight [16]. Chronic kidney disease was defined as the baseline estimated glomerular filtration rate (eGFR) < 60 mL/min/1.73 m^2^, which was calculated using the simplified Modification of Diet in Renal Disease formula [17]. Septic shock was identified based on the Sepsis-3 definitions [1]. Acute kidney injury was determined according to the Acute Kidney Injury Network criteria [18].

### 2.4. Outcomes Measurement

The primary outcome of this study was to investigate the association between RDW and adverse outcomes in patients with sepsis. We included ICU admission, endotracheal intubation (respiratory failure), septic shock (hypotension requiring vasopressor support, and a serum lactate level > 2 mmol/L), acute kidney injury, and 30-day in-hospital mortality risk as adverse outcomes. The secondary outcome was to compare the mortality discriminative ability between lactate and RDW levels.

### 2.5. Statistical Analysis

All data were analyzed using the Statistical Package for the Social Sciences version 22 (SPSS Inc., Chicago, IL, USA) and MedCalc version 18.2.1 software. Data were expressed as means with standard deviations or medians with interquartile ranges for continuous variables, and numbers (%) for categorical variables. The differences between the normal and high RDW groups were compared using the two-sample t-test and chi-square test (or Fisher’s exact test) for continuous and categorical variables, respectively. The Mann–Whitney test was used for continuous variables if data were not normally distributed. Univariate analysis was used to establish potential variables associated with 30-day in-hospital mortality risk in patients with sepsis. All variables with a *p*-value < 0.1 in the univariate analysis were included in the multivariate logistic regression model to analyze independent factors. Age and sex were mandatory variables in the model, irrespective of the *p*-value in the univariate analysis. The discriminative ability in predicting the risk of mortality of these independent factors using the area under the receiver operating characteristic (ROC) curves was tested. The Delong method was used to compare the area under the curve (AUC) of the studied variables [19]. Statistical significance was set at a two-tailed *p*-value < 0.05.

## 3. Results

**Figure 1:**
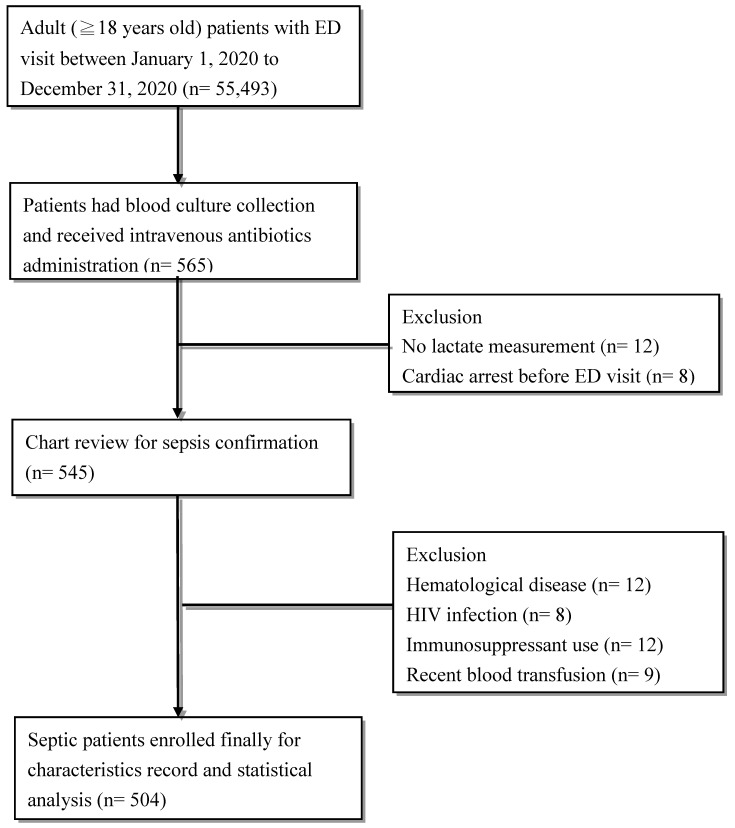
Flowchart of patient enrollment.

As shown in Figure 1, 565 of 55,493 adult patients who visited the ED during the study period were selected. After excluding patients with no data on lactate measurement (*n* = 12), a cardiac arrest event before ED visit (*n* = 8), known hematological disease (*n* = 12), HIV infection (*n* = 8), immunosuppressant use (*n* = 12), and those who received recent blood transfusion (*n* = 9), 504 patients with sepsis were enrolled for further analysis.

### 3.1. Baseline Characteristics

As shown in Table 1, the mean patient age was 68.1 ± 15.8 years, and most (61.9%) patients were males. Approximately three-quarters (72.2%) of the patients met the SIRS criteria; the mean SOFA score was 8 ± 2, indicating relatively high disease severity. Regarding the source of sepsis, respiratory tract infection (48.2%) was the leading cause of sepsis, followed by intra-abdominal (20.2%) and urinary tract (15.9%) infections. Approximately 50% of patients had comorbidities, such as diabetes mellitus (47.4%), hypertension (56.3%), and chronic kidney disease (58.5%). Patients in the high RDW group had significantly higher SOFA score (9 ± 2 vs. 7 ± 2, *p* < 0.01) and lower hemoglobin (10.3 ± 2.6 g/dL vs. 12.4 ± 2.3 g/dL, *p* < 0.01) levels than the normal RDW group. There were no significant differences in lactate or C-reactive protein (CRP) levels between the two groups (Table 1).

### 3.2. Outcomes Analysis

As shown in Table 2, the overall 30-day in-hospital mortality rate was 27.2% (137/504). The high proportion of ICU admission, endotracheal intubation, septic shock, and acute kidney injury was in line with their disease severity. Notably, the patients in the high RDW group had a significantly higher rate of ICU admission (48.8% vs. 32.4%, *p* = 0.03), septic shock (39.2% vs. 23.5%, *p* < 0.01), and 30-day in-hospital mortality (32.0% vs. 20.7%, *p* < 0.01).

Next, we examined the factors associated with 30-day in-hospital mortality risk in patients with sepsis. We chose the variables that revealed significant differences between survivor and non-survivor status in our regression model. We excluded patients with chronic kidney disease due to its collinearity with the SOFA score. As shown in Table 3, the SOFA score (hazard ratio (HR) = 3.21, *p* < 0.01), serum RDW (HR = 1.05, *p* < 0.01) and lactate (HR = 1.06, *p* < 0.01) remained independent factors associated with mortality risk in patients with sepsis.

**Figure 2:**
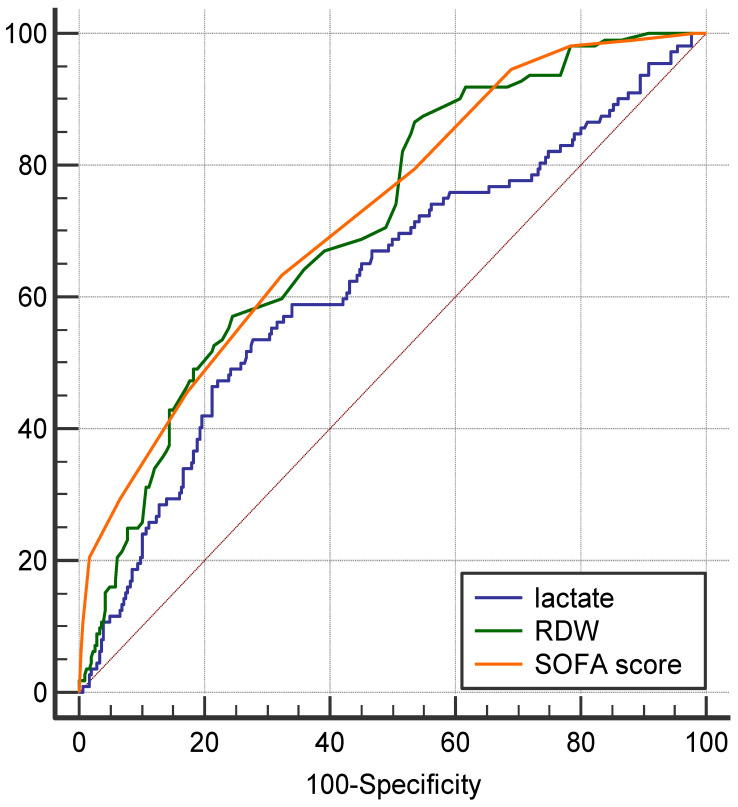
Receiver operating characteristic curves for 30-day in-hospital mortality predicting ability of SOFA score, initial serum lactate, and RDW levels in patients with sepsis.

We further compared the 30-day in-hospital mortality discriminative ability of the three independent parameters using ROC curves. As shown in Figure 2, the AUC of the three parameters was as follows: RDW, 0.71 (95% confidence interval [CI] = 0.67–0.76, *p* < 0.01), SOFA score, 0.73 (95%CI = 0.68–0.77, *p* < 0.01), and lactate, 0.63 (95%CI = 0.57–0.67, *p* < 0.01). Furthermore, the AUC of RDW was significantly higher than that of lactate (*p* = 0.02), and similar to the SOFA score (*p* = 0.72).

## 4. Discussion

In this ED-based, single-centered, retrospective study, we validated the prognostic significance of RDW in patients with sepsis. We demonstrated that high RDW in septic patients was not only associated with an increased risk of 30-day in-hospital mortality, but also with septic shock development and ICU admission. Moreover, SOFA score, serum lactate, and RDW levels were independent factors associated with mortality risk in septic patients, and RDW revealed superior mortality discriminative ability compared to lactate.

The exact pathophysiology of elevated RDW in sepsis is not well understood; however, it is possibly related to a systemic inflammatory response, leading to high levels of pro-inflammatory cytokines (e.g., interleukin-6, tumor necrosis factor-α) and increased oxidative stress in sepsis syndrome [20]. This inflammatory response negatively affects bone marrow function, alters RBCs half-life, decreases erythropoietin production and iron metabolism, increases hemolysis, resulting in ineffective production or increased destruction of RBCs and, further increases in the size heterogeneity of RBCs [21]. Increased oxidative stress reduces RBCs survival and increases immature RBCs into the peripheral circulation, leading to elevated RDW [10]. Furthermore, the glycoproteins and ion channels of the membrane in RBCs alterations in sepsis can produce RBCs morphology changes [22]. The elevated RDW or presence of frailty also reflects the reduced physiological reserve, further associated with mortality risk in patients with sepsis. Taken together, the elevation of RDW represents multiple harmful pathological processes, including excessive oxidative and inflammatory stress, all of which occur simultaneously in critical illness and result in mortality [5,10].

It is not surprising that patients in the high RDW group had significantly lower hemoglobin levels than those in the normal RDW group, since elevated RDW is associated with abnormal hemoglobin production or hemolysis, leading to a low number of RBCs [23]. RDW was developed in conjunction with MCV to differentiate the causes of anemia and is particularly useful in evaluating patients with chronic diseases or nutritional-deficiency related anemia decades ago [24]. Moreover, the significantly higher SOFA score in the high RDW group can be attributed to impaired microcirculation and organ ischemia or reduced physiological reserve, resulting in higher severity of organ dysfunction [25].

Our results demonstrated that the respiratory tract accounted for the most common site of infection as well as a higher risk of mortality in patients with sepsis, which was compatible with a recent US nationwide study among hospitalizations of patients with sepsis stratified by infection sites [26]. Higher mortality risk in septic patients with chronic kidney disease in our regression model was also in line with septic studies regarding the effect of comorbidities on mortality outcomes and also, with leukocyte dysfunction and decreased inflammatory cytokines clearance, which contribute to immune dysfunction and mortality events in patients with renal function insufficiency [27,28].

Except for the risk of mortality, RDW has never been validated for other adverse outcome associations in patients with sepsis. The significantly higher risk of septic shock development and ICU admission in our high RDW group can be explained by their high oxidative stress, red cell apoptosis and peripheral phagocytosis activation, reactive oxygen species generation, further precipitating vital organs and circulatory dysfunction that requires vasopressor support and intensive care [29]. The advanced age and a high proportion of comorbidities in our patients also made them more susceptible to infection and had a higher risk of adverse outcomes with sepsis [30].

RDW was found to have a moderate correlation with lactate levels in critically ill pediatric patients [12]. Hyperlactatemia in sepsis is based on the theory that inadequate oxygen delivery causes tissue damage and anaerobic glycolysis, although recent evidence has revealed that a more complicated mechanism including adaptive metabolic processes, comorbidities, and drug/toxins all contribute to lactate accumulation [31]. Nevertheless, hyperlactatemia is still correlated with mortality in patients with septic shock and is a widely used prognostic marker [2]. Determination of serum lactate level may help assessment of hypoxia and lactic acidosis in patients with sepsis or septic shock [32]. More importantly, lactate levels should be measured in a timely manner; otherwise, it would be artificially elevated as red blood cell metabolism continues [3,31]. Compared with lactate, RDW is a simple and rapidly obtained parameter included in the complete blood count panel, which demonstrated a superior prognosis discrimination ability in our study.

Our study had several limitations. First, this was a retrospective study, which made recall and selection bias inevitable; the ED-based, single-centered nature may have limited the extrapolation potential of these results. Second, nutritional status of sepsis patients was not considered, including iron, folate, and vitamin B12 levels. We also did not report the reticulocyte count and blood smear results of our patients, which may have influenced their RDW levels [24]. Finally, since the mechanism between sepsis-related mortality and increased RDW remains not fully understood, we may have missed some unknown confounders in this study.

## 5. Conclusions

In conclusion, high RDW in septic patients was associated with an increased risk of 30-day in-hospital mortality, septic shock, and ICU admission. Moreover, RDW showed superior mortality discriminative ability in compared to lactate. Patients with sepsis and high RDW levels may need more intensive care, such as early vasopressor support and hemodynamic status monitor. Clinicians can utilize this simple, rapid, and inexpensive parameter as a prognostic marker in patients with sepsis, and aid in earlier recognition of patients with a high risk of mortality who need prompt management. Future larger prospective studies in combination of sepsis management bundle and RDW levels are warranted to confirm these findings.

## Figures and Tables

**Table 1:** Baseline characteristics of patients with sepsis based on their initial red cell distribution width (*n* = 504).

Characteristics	All (*n* = 504)	Normal RDW ≤14.5% (*n* = 213)	High RDW >14.5% (*n* = 291)	*p* Value
Age, y, mean ± SD	68.1 ± 15.8	68.3 ± 14.7	68.0 ± 16.6	0.86
Male, *n* (%)	312 (61.9)	135 (63.4)	177 (60.8)	0.56
SIRS, *n* (%)	364 (72.2)	147 (69.0)	217 (74.6)	0.19
qSOFA score, *n* (%)				0.11
0	78 (15.5)	38 (17.8)	40 (13.7)	
1	164 (32.5)	75 (35.2)	89 (30.6)	
2	157 (31.2)	61 (28.6)	96 (33.0)	
3	105 (20.8)	39 (18.3)	66 (22.7)	
SOFA score, mean ± SD	8 ± 2	7 ± 2	9 ± 2	<0.01 *
Frailty syndrome, *n* (%)	62 (12.3)	27 (12.7)	35 (12.0)	0.84
Source of sepsis, *n* (%)				
Respiratory tract infection	243 (48.2)	109 (51.2)	134 (46.0)	0.26
Urinary tract infection	80 (15.9)	32 (15.0)	48 (16.5)	0.66
Intra-abdominal infection	102 (20.2)	46 (21.6)	56 (19.2)	0.52
Soft tissue infection	33 (6.5)	9 (4.2)	24 (8.2)	0.10
Other	46 (9.1)	17 (8.0)	29 (10.0)	0.53
Comorbidities, *n* (%)				
Diabetes Mellitus	239 (47.4)	94 (44.1)	145 (49.8)	0.21
Hypertension	284 (56.3)	128 (60.1)	156 (53.6)	0.15
Coronary artery disease	101 (20.0)	48 (22.5)	53 (18.2)	0.23
Chronic kidney disease	295 (58.5)	123 (57.7)	172 (59.1)	0.76
Cerebrovascular accident	56 (11.1)	30 (14.2)	26 (8.9)	0.09
Dyslipidemia	60 (11.9)	25 (11.7)	35 (12.0)	1.00
Laboratory results				
Hemoglobin, g/dL, mean ± SD	11.1 ± 2.6	12.4 ± 2.3	10.3 ± 2.6	<0.01 *
Leukocyte, × 10^9^/L, median (IQR)	12.7 (7.5–17.8)	12.2 (7.1–16.9)	13.1 (8.1–18.8)	0.18
Creatinine, mg/dL, median (IQR)	1.8 (1.2–2.8)	1.7 (1.2–2.6)	1.9 (1.2–3.0)	0.14
CRP, mg/dL, median (IQR)	107.8 (43.7–197.8)	105.6 (44.2–195.2)	111.9 (37.9–203.8)	0.69
Lactate, mmol/L, median (IQR)	2.3 (1.5–4.2)	2.2 (1.5–4.0)	2.4 (1.4–4.5)	0.57

* *p* < 0.05. SD: standard deviation. IQR: interquartile range. SIRS: systemic inflammatory response syndrome. qSOFA: quick sepsis-related organ failure assessment. SOFA: sepsis-related organ failure assessment. CRP: c-reactive protein.

**Table 2:** Outcome analysis of patients with sepsis based on their initial red cell distribution width (*n* = 504).

Variables, *n* (%)	All (*n* = 504)	Normal RDW ≤14.5% (*n* = 213)	High RDW >14.5% (*n* = 291)	*p* Value
Intensive care unit admission	211 (41.9)	69 (32.4)	142 (48.8)	0.03 *
Endotracheal intubation	180 (35.7)	65 (30.5)	115 (39.5)	0.19
Septic shock	164 (32.5)	50 (23.5)	114 (39.2)	<0.01 *
Acute kidney injury	175 (34.7)	69 (32.4)	106 (36.4)	0.48
30-day in-hospital mortality	137 (27.2)	44 (20.7)	93 (32.0)	<0.01 *

* *p* < 0.05.

**Table 3:** Univariate and multivariate logistic regression models of factors associated with 30-day in-hospital mortality in patients with sepsis (*n* = 504).

Variables	Univariate		Multivariate	
	HR (95%CI)	*p* Value	HR (95%CI)	*p* Value
Age (year)	1.01 (0.99–1.02)	0.11	1.01 (0.99–1.03)	0.17
Sex (male)	1.43 (0.94–2.16)	0.09	1.76 (0.96–3.23)	0.07
Chronic kidney disease	1.81 (1.19–2.74)	<0.01 *		
Respiratory tract infection	1.55 (1.05–2.31)	0.03 *	1.21 (0.74–1.90)	0.98
Urinary tract infection	0.52 (0.28–0.96)	0.04 *	0.64 (0.24–1.34)	0.39
SOFA score	3.17 (2.55–3.95)	<0.01 *	3.21 (2.55–4.03)	<0.01 *
RDW (%)	1.16 (1.08–1.25)	<0.01 *	1.05 (1.02–1.08)	<0.01 *
lactate	1.08 (1.03–1.14)	<0.01 *	1.06 (1.02–1.11)	<0.01 *

* p < 0.05. HR: hazard ratio. CI: confidence interval. SOFA: sepsis-related organ failure assessment.

## Data Availability

The datasets generated and analyzed during the current study are available from the corresponding authors upon reasonable request.
